# Neurosarcomatous amelanotic transformation of malignant melanoma presenting as malignant periopheral nerve sheath tumor: Rare case report

**DOI:** 10.1097/MD.0000000000034034

**Published:** 2023-06-23

**Authors:** Lu Bofan, Xiu Xiaofei, Zhang Jingwen, Zhang Zuzhuo, Ma Tianxiao, Gao Feng, Zhang Guochuan, Zhuang Zhou

**Affiliations:** a Clinical medicine of Basic Medical College, HeBei Medical university, Shijiazhuang, Hebei, P. R. China; b Department of Pathology, The Third Affiliated Hospital of Hebei Medical University, Shijiazhuang, Hebei, P. R. China; c Department of Neurosurgery, The Second Hospital of Hebei Medical University, Hebei Medical University, Shijiazhuang, Hebei, China; d Department of Radiology, The Third Hospital of Hebei Medical University, Shijiazhuang, China; e Department of Orthopedic Oncology, Third Hospital of Hebei Medical University, Shijiazhuang, China.

**Keywords:** case report, malignant melanoma, malignant periopheral nerve sheath tumor, metastatic, pathology

## Abstract

Malignant melanoma (MM) is notorious for its remarkable morphological variation and aberrant histopathological patterns. In addition, Malignant Periopheral Nerve Sheath Tumor (MPNST) is an uncommon but aggressive soft tissue sarcoma. Because of the common embryological origin of melanocytes and Schwann cells in the neural crest, discriminating between a particular type of MM and MPNST can be difficult, particularly when they are amelanotic. Our goal is to increase awareness among clinicians of the rare variations of MM and the importance of medical history in improving the accuracy of the final clinical diagnosis.

**Patient concerns:**

A 68-year-old man was admitted to the hospital due to pain in his right ankle, which had persisted for 8 months, along with swelling for 4 months. Medical history revealed delayed healing of right plantar for 5 years after a traumatic injury.

**Diagnoses:**

The ankle mass was initially diagnosed as MPNST through biopsy. After reviewing the patient’s medical history and receiving the final pathological report following amputation, we have revised the diagnosis to metastatic amelanotic desmoplastic melanoma in the ankle part and lentigo maligna melanoma in the plantar part. This is due to both lesions displaying positive markers or mutated genes in immunohistology and Gene Mutation Detection, indicating homology between the 2 tumors.

**Interventions:**

Due to the malignant characteristics of the tumor and the patient’s wishes, amputation of the right lower leg was carried out.

**Outcomes:**

Subsequently, the patient was treated with interferon-γ and immunosuppressant PD-1 inhibitor, and survived for 1 year after amputation.

**Lessons:**

Clinical data, immunohistochemisty biomarkers and genes detection results can serve as valuable evidence for pathologists and clinicians in identifying the disease process. Collaborative efforts between clinicians and scientists are crucial in order to identify specific markers that can effectively differentiate between the 2 tumors, thereby enhancing the conclusiveness of the diagnosis.

## 1. Introduction

Malignant melanoma (MM) is a tumor produced by the malignant transformation of melanocytes, which originated from the neural crest.^[1]^ MM is very aggressive, and has a wide range of histologic variants and cytomorphologic features.^[2]^ It can metastasize in an early stage of the disease and the patient can be presented with metastatic lesions as the main manifestation.^[3]^ A more serious situation is that about 4% of patients with unknown primary.^[4]^ Sometimes, the specific type of metastatic lesions of MM is difficult to be clearly diagnosed even with multiple detection methods.^[5]^ Thus, misdiagnosis of amelanotic melanoma is still a notable factor contributing to diagnostic errors, earning this lesion the nickname of “the great masquerader.”^[6]^ We present a rare case in which the patient initially complained of ankle symptoms and was diagnosed with malignant periopheral nerve sheath tumor (MPNST) based on an ankle biopsy conducted at a local hospital. After undergoing surgery and a comprehensive evaluation that included immunohistochemistry (IHC) and gene mutation detection, the patient’s medical history was also carefully reviewed, ultimately resulting in a revised diagnosis.

## 2. Case report

### 2.1. Clinical course (chief complaints and physical examinations, imaging examinations and final diagnosis, treatment, outcome and follow-up)

**Figure 1.**
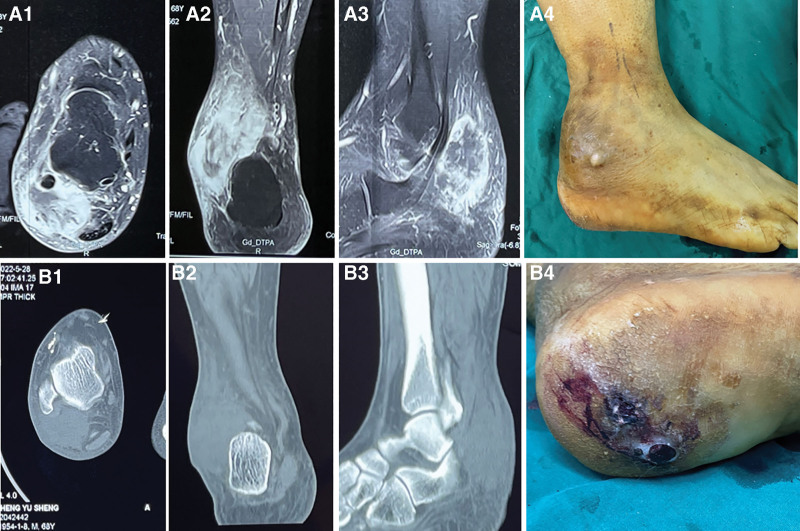
Displays the CT and MRI examinations as well as the appearance of the lesions. A1, A2, and A3 show the transverse, coronal, and sagittal MRI views of the ankle part tumor, respectively. B1, B2, and B3 show the transverse, coronal, and sagittal MRI views of the plantar part tumor, respectively. A4 and A5 depict the appearance of the tumors in the ankle and plantar regions, respectively.

**Figures 2.**
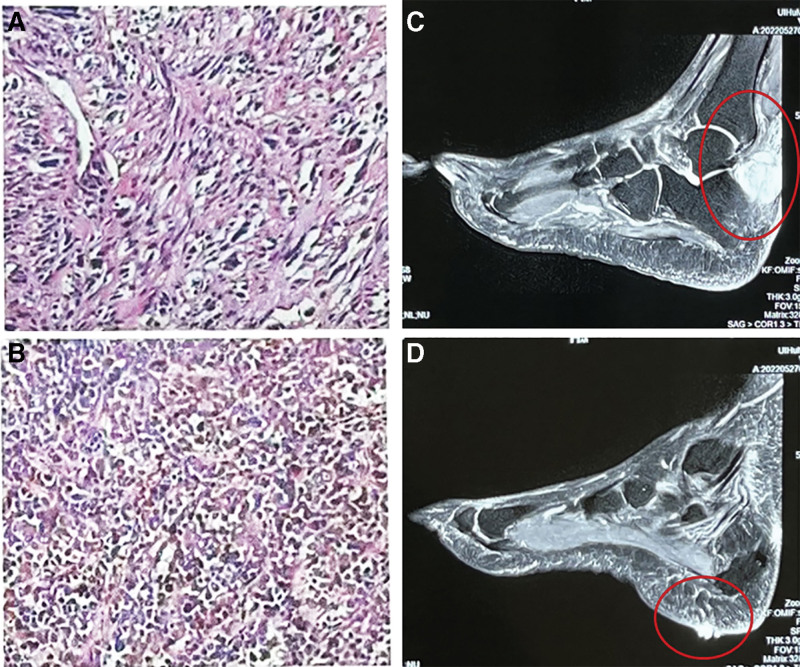
A and 2B show the H&E sections of the tumor in the ankle and plantar regions, respectively. MRI images (Fig. 2C and D) indicated that the lesions in the patient’s right ankle and right plantar were separate.

A 68-year-old man was admitted to the hospital due to pain in his right ankle, which had persisted for 8 months, along with swelling for 4 months. The patient’s right toe movement is limited. The patient’s medical history revealed delayed healing of right plantar for 5 years after a traumatic injury. An ulcer had developed on the sole of his right foot, accompanied by a small amount of pus (Fig. 1). CT and MRI scans showed that lesions in his right ankle and right plantar were separate. Then, puncture biopsy of the right ankle tumor was performed at local hospital and diagnosed as mesenchymal tissue tumor, furthermore, the result of a pathological consultation amended the diagnosis to MNPST. PET/CT revealed 2 hypermetabolic lymph nodes in the right groin, suggestive of metastasis. All other examinations, including lung, brain, abdomen, were normal at the time of initial diagnosis. General physical examination, and routine blood tests were also normal. The patient had a history of thyroid cancer, which was treated with thyroidectomy 10 years ago, and there was no recurrence during follow-up. He also had a history of coronary arteriosclerosis for 5 years. Due to the malignant characteristics of the tumor and the patient’s wishes, amputation of the right lower leg was carried out. The patient refused to have a biopsy or resection of the inguinal lesions. Final pathology report revealed 2 types of tumors: lentigo maligna melanoma in the plantar part and metastatic desmoplastic melanoma (DM) in the ankle part (Fig. 2). Subsequently, the patient was treated with interferon-γ and immunosuppressant PD-1 inhibitor, and survived for 1 year after amputation.

### 2.2. General manifestations and histology

The Plantar Part: there is a 3 × 2 cm black-gray nodular area on the right plantar, accompanied by skin ulcer. Microscopic examination revealed the entire epidermis was absent, with neutrophil exudation and cellulose deposition on the surface of the tumor. The tumor invaded the dermis reticular layer as an enlarged nodule with clear boundaries with surrounding tissues. We have classified the tumor as Clark grade IV, Breslow thickness 3.4 mm. The tumor cells are epithelioid, rich in cytoplasm, eosinophilic, and have polymorphous vesicular nuclei with nucleoli, eosinophilic, mitotic count 7/mm2, and Ki-67 up to 40%. The tumor is accompanied by excessive melanin deposition inside and outside the cells. A few lymphocytes infiltrated around the tumor, but the lymphocytes did not invade the tumor tissue. There is no microsatellite metastasis, satellite metastasis, intravascular tumor thrombus, or nerve invasion.

The Ankle Part: Above the plantar lesion by 4 cm, a 5 × 4 × 4cm gray-white mass can be seen at the right ankle. The section is grayish white, hard, and has a clear boundary with surrounding soft tissues, and it invades subcutaneous adipose tissue. The surface of the tumor has normal skin appearance, without pigmented skin lesions, skin color or erythematous nodules or sclerotic plaques. Microscopic examination showed that the tumor was located in the dermis reticular layer, the tumor cell body was elongated with weakly eosinophilic cytoplasm. The nucleus was long spindle-shaped, cigar-shaped, and vesicular, deeply stained, accompanied by interstitial fibrosis and a large amount of collagen formation. In some areas, the cytoplasm boundary of tumor cells is unclear, eosinophilic, the nucleus is small, irregular wavy, comma-shaped, similar to MNPST. The tumor area with Schwann cell differentiation can be seen with multifocal perineural infiltration. Some spindle tumor cells are arranged in a striped pattern, with marked cell atypia, polymorphous nuclei, and multinuclear tumor giant cells and pathological mitosis. No pigment deposition was found in tumor cells or between cells.

The skin on the area between the right foot and ankle appears normal with no pigmented skin lesions, abnormal skin color, erythematous nodules, or sclerotic plaques. Full tissue sampling and sectioning between the lesions revealed hyperkeratosis of the epidermis but no melanomas or nevus-like lesions were detected in the epidermis, dermis, or subcutaneous tissues.

### 2.3. Immunohistology and gene mutation detection

Paraffin-embedded and frozen sections were stained using the APAAP technique with various antibodies, all of which were purchased from Maxim, Fuzhou, China. The immunohistochemistry analysis of the right plantar tumor showed diffuse positivity for SOX-10, HMB45, Melan-A, with weak S100 positivity in certain areas. PD-L1 was negative with a Tumor Proportion Score <70%. In the right ankle tumor, S100 and SOX-10 were diffusely positive in spindle tumor cells, while HMB45 and Melan-A were negative. PD-L1 was positive with a tumor proportion score >70%. Notably, a small epithelioid tumor area in the ankle lesions exhibited similar histological morphology to that of the right plantar, but without pigment deposition. However, the expression of immunohistochemical markers differed from the right foot tumors, with negative expression of S100, SOX-10, HMB45, and Melan-A.

A genetic panel comprising 525 genes was used to perform a comprehensive test of genetic variation on both parts of the tumor separately shown in Table S1, Supplemental Digital Content, http://links.lww.com/MD/J139 and Table S2, Supplemental Digital Content, http://links.lww.com/MD/J140. The right ankle tumor exhibited 14 gene mutations, while the right plantar tumor had 5 gene mutations. NRAS and NSD2 were 2 identical mutant genes found in both parts of the tumor, with the same mutation style. In addition, 3 gene mutations occurred only in the right plantar tumor with 3 different mutation styles, whereas 11 gene mutations were found exclusively in the right ankle tumor, with 4 genes deletion and 7 genes missense mutation. The mutation abundance in the right ankle tumor was almost double that of the right plantar tumor. Furthermore, the exclusive gene abundance in the right ankle tumor varied widely, with 4 genes (TP53, MYCN, PTEN, and TCF7L2) exhibiting higher abundance, and 7 genes (AKT1, RASA1, KDR, IGF2R, FANCE, APOB, and AMER1) displaying lower abundance, as shown in Table 1.

**Table 1:** Genetic variation results of right plantar tumor and right ankle tumor.

	Right plantar tumor		Right ankle tumor	
	Gene variation type	Mutation abundance	Gene variation type	Mutation abundance
NRAS	p.Q61R (C.182A > G)	15.51%	p.Q61R (C.182A > G)	31.75%
NSD2	p.S228F (c.683C > T)	9.27%	p.S228F (c.683C > T)	18.88%
ARID2	p.V702Sfs*56 (c.2103delT)	9.28%	-	-
PRKDC-SPIDR	Fusion	8.77%	-	-
BRAF	p.V681A (c.2042T > C)	8.26%	-	-
TP53	-	-	p.Q16Hfs*26 (c.48_54delGGAAACA)	36.14%
MYCN	-	-	p.E443* (c.1327G > T)	17.63%
PTEN	-	-	p.R47S (c.141G > T)	33.16%
TCF7L2	-	-	p.E29del (c.85_87delGAG)	28.56%
AKT1	-	-	p.K420del (c.1258_1260delAAG)	1.81%
RASA1	-	-	p.L770del (c.2307_2309delGTT)	1.72%
KDR	-	-	p.R946C (c.2836C > T)	1.49%
IGF2R	-	-	p.K482R (c.1445A > G)	1.3%
FANCE	-	-	p.P110T (c.328C > A)	1.27%
APOB	-	-	p.S671R (c.2011A > C)	1.22%
AMER1	-	-	p.F698C (c.2093T > G)	1.13%

## 3. Discussion

This rare case describes our experience with an elderly patient who admitted to the hospital with initial diagnosis of ankle MPNST and had been scheduled for amputation. It was found after imaging examination that the ankle part of the tumor and the plantar part of the tumor were in close proximity but not contiguous. Based on the patient’s medical history, histopathological examination, immunohistochemistry, and even gene detection, it was determined that there was a correlation between the 2 parts tumors, ultimately leading to a revised diagnosis of ankle metastatic amelanotic MM, that is metastatic desmoplastic melanoma.

MM is known for its wide range of histologic variants and cytomorphologic features, which can mimic various poorly differentiated carcinomas like neuroendocrine tumors, germ cell tumors, and different types of soft tissue sarcomas.^[7–11]^ Moreover, The primary and metastatic MM can also assume different histological patterns, making the light-microscopic identification of the tumors difficult.^[12–14]^ And metastases from a primary cutaneous MM of the classical type may present a divergent histological appearance.^[15]^ A Minagawa^[15]^ reported diagnosis of a nonpigmented red ulcerative nodule was detected on his right upper back, which was suspected to be an amelanotic cutaneous metastasis of melanoma based on the dermoscopic findings. Akash Agarwal^[16]^ report an rare case with primary pigmented MM to metastatic amelanotic variant, which should arouse the attention of clinical doctors. In such instances, identifying the melanocytic nature of a metastatic tumor can be challenging, especially if the primary tumor is unknown, which occurs in approximately 5% of all cases of MM.^[17,18]^ P. Lodding^[19]^ reported 7 cases that resembled MPNST or monophasic synovial sarcoma. And Danielle Verver^[20]^ noted that patients with Melanoma of unknown primary typically exhibit poorer prognostic characteristics than those with melanoma of known primary. Accurate diagnosis is essential for clinical doctors to provide appropriate treatment and improve the prognosis and quality of life for patients.

Several cases of metastatic MMs simulating sarcomas are on record, including cases of MM mimicking monophasic synovial sarcoma, fibrosarcoma, malignant fibrous histiocytoma, dermatofibrosarcoma, haemangiopericytoma, primitive neuroectodermal tumor, and malignant peripheral nerve sheath tumor (MPNST).^[2,19,21]^ Because of the common embryological origin of melanocytes and Schwann cells in the neural crest,^[22]^ discrimination between MM and MPNST on the basis of histological, immunohistochemical criteria can pose extremely difficulties. Klaus Jürgen Schmitz reported^[23]^ 42-year-old female patient with right cervical swelling, which initial diagnosis with MM by biopsy. Further examination including IHC and Electron micrograph allowed the revision of the diagnosis to that of melanotic schwannoma. D Schadendorf^[24]^ also reported a rare case with a 78-year-old woman presented with a nodule on the sole of her left foot, histological, immunochemical and ultrastructural studies of the primary tumor revealed melanocytic and Schwannian characteristics, and posed diagnostic difficulties. The final diagnosis of a MM with Schwannian differentiation was established on the basis of the clinical course, with the development of metastases in the subcutis, lymph nodes, liver and brain, as well as a shift in differentiation of the metastases towards cells containing giant melanosomes, typical of melanoma.

DM is a rare type of melanoma, accounting for <4% of all melanomas, and is considered a variant of spindle cell melanoma.^[25]^ Clinicians and pathologists face challenges in diagnosing DM due to several limitations, including nonspecific and often banal appearance of DM.^[26]^ Moreover, there are no accepted diagnostic criteria for a MPNST.^[27]^ Because both tumor cells are derived from neural crest, the morphology of DM closely resembles MPNST. Sometimes, MPNST are problematic to diagnose due to the morphological overlap with DM and the lack of specific IHC or genetic alterations.^[28]^ The most challenging differential diagnosis is between DM and cutaneous MPNST since both neoplasms show S100 positivity (mainly the epithelioid variant of MPNST) and can lack IHC markers of melanocytic differentiation.^[28]^ With regards to the oncological management and prognosis of DM and MPNST, a correct diagnosis is very important since both DM and MPNST require wide excision with 1 to 2 cm margins. MPNST can be treated with surgery or combined with adjuvant chemotherapy.^[29]^ In contrast, conventional melanoma requires sentinel lymph node biopsy for further therapeutic management, interferon-γ or and immunosuppressant PD-1 inhibitor treatment is often required.^[30–32]^

In our case, the patient was admitted to the hospital based on an ankle biopsy that was diagnosed as MPNST, but upon further examination of the patient’s medical history and final pathological results after amputation, the diagnosis was revised to lentigo maligna melanoma of the right plantar with metastasis to the right ankle, classified as Desmoplastic Melanoma. The immunohistochemistry results show that both tumors express SOX100 and S100 proteins, indicating that these 2 tumors are of the same origin. positive HMB45 and Melan-A in the plantar confirmed the diagnosis with malignant melanoma, while the ankle tumor is negative for HMB45 and Melan-A. The comprehensive results suggest that transformation of ankle tumors towards a DM direction should be considered.^[33]^ Additionally, negative results for Desmin, CD56, Myogenin, and MyoD1 can exclude diagnoses such as leiomyoma, rhabdomyoma, primitive neuroectodermal tumor, and lymphoma. The IHC results showed an increase in KI-67 in metastatic lesions, indicating a stronger and more active proliferative ability of tumor cells in metastatic lesions. Multiple genes involved in cell cycle regulation, division, and growth have mutated in metastatic lesions, and the number and abundance of mutated genes are higher than those in the primary lesion. The 2 mutation sites (NRAS and NSD2) with the highest abundance in the primary lesion are also highly abundant in the metastatic lesion, indicating once again that the tumors in both sites are of the same origin. However, there were also differences in other markers or genes, suggesting differentiation or transformation in the metastatic tumors compared to the primary site.

Since both melanocytes and Schwann cells arise from the neural crest, it was previously hypothesized by Masson^[34]^ that naevus cells in the upper dermis originate from melanocytes, whereas those in the lower dermis originate from Schwann cells. Thus, there are definitely some differences here, which we may not have discovered yet. The diagnosis of this rare case was accomplished through a combination of clinical data, and comparisons with similar IHC biomarkers and genes. However, if there are specific IHC biomarkers and genetic phenotypes available, the diagnosis of this case will be more conclusive. This is the limitation of this case and the direction for future efforts.

To conclusion, this case exemplifies the process of diagnosis correction by pathologists and clinical doctors. For 2 different malignant tumors with the same or similar origin, and their treatment plans and prognosis are very different, it is crucial to identify specific patient case types to guide clinical decision. For rare tumors such as this 1, more efforts need to be made to verify new immunohistochemical markers or gene mutation sites, and carefully collect patient clinical data to gather evidence on past tumor behavior in order to determine the nature and source of the tumor.

## Author contributions

**Conceptualization:** Zhang Jingwen, Zhuang Zhou.

**Formal analysis:** Zhuang Zhou.

**Methodology:** Zhuang Zhou.

**Resources:** Zhuang Zhou.

**Supervision:** Zhang Jingwen, Zhang Guochuan, Zhuang Zhou.

**Validation:** Xiu Xiaofei, Zhang Jingwen, Zhang Guochuan, Zhuang Zhou.

**Writing – original draft:** Lu Bofan, Xiu Xiaofei, Zhang Zuzhuo, Ma Tianxiao.

**Writing – review & editing:** Gao Feng, Zhang Jingwen, Zhang Guochuan.

## Supplementary Material




