# Supplementary material for: Neurosarcomatous amelanotic transformation of malignant melanoma presenting as malignant periopheral nerve sheath tumor: Rare case report
Source: Medicine (Baltimore). 2023 Jun 23;102(25):e34034. doi: 10.1097/MD.0000000000034034 (PMC10289641; doi:10.1097/MD.0000000000034034)
Supplement: Supplementary file 1 [file medi-102-e34034-s001.pdf]

Table 1 Genetic Variation List

CB1	CCND2	DSCAM	FGF3	HNF1A	MAP3K13	NHEJ1	POT1	RXRA	TAP1
ABCG2	CCND3	DUSP4	FGF4	HOXB13	MAP3K14	NKX2-1	POU5F1	RYBP	TAP2
ABL1	CCNE1	DUT	FGF6	HRAS	MAP4K3	NKX3-1	PPARG	RYR2	TBL1XR1
ABRAXAS1	CD74	DYNC2H1	FGF10	HSD3B1	MAPK1	NLRP1	PPM1D	RYR3	TBX3
ACSL3	CD79B	E2F3	FGF12	HSD17B4	MAPK3	NOTCH1	PPP2R1A	SCG5	TCF3
ACVR1	CD274	EDC4	FGF14	HSP90AA1	MAPKAP1	NOTCH2	PPP2R2A	SDC4	TCF4
ACVR2A	CD276	EGFR	FGF19	HSPA4	MAX	NOTCH3	PPP4R2	SDHA	TCF7L2
ACYP2	CDC27	EIF1AX	FGFR1	ICOSLG	MB21D2	NOTCH4	PPP6C	SDHAF2	TEK
ADGRA2	CDC42	EIF4A2	FGFR2	ID3	MC1R	NPM1	PRDM1	SDHB	TERT
AFF4	CDC73	ELAC2	FGFR3	IDH1	MCL1	NQO1	PRDM14	SDHC	TET1
AJUBA	CDH1	ELF3	FGFR4	IDH2	MDC1	NR4A3	PREX2	SDHD	TET2
AKT1	CDH9	ELOC	FH	IFNGR1	MDH2	NRAS	PRKAR1A	SEMA3C	TFE3
AKT2	CDK4	EME1	FLCN	IGF1	MDM2	NRG1	PRKCI	SESN1	TGFBR1
AKT3	CDK6	EME2	FLI1	IGF1R	MDM4	NSD1	PRKD1	SESN2	TGFBR2
ALK	CDK8	EML4	FLNA	IGF2	MECOM	NSD2	PRKDC	SESN3	TIPARP
AMER1	CDK12	EMSY	FLT1	IGF2R	MED12	NSD3	PRKN	SETD2	TMEM127
APC	CDKN1A	EP300	FLT3	IKBKE	MEF2B	NT5C2	PRPF40B	SF3B1	TMPRSS2
APOB	CDKN1B	EPCAM	FLT4	IKZF1	MEN1	NTHL1	PRSS1	SGK1	TNFAIP3
AR	CDKN1C	EPHA2	FOXA1	IL7R	MERTK	NTRK1	PTCH1	SH2B3	TNFRSF14
ARAF	CDKN2A	EPHA3	FOXL2	IL10	MET	NTRK2	PTCH2	SH2D1A	TNFSF11
ARID1A	CDKN2B	EPHA4	FOXO1	INHA	MGA	NTRK3	PTEN	SHOC2	TOP1
ARID1B	CDKN2C	EPHB1	FOXP1	INHBA	MGMT	NUDT18	PTGIS	SHPRH	TOP3A
ARID2	CDRT4	EPPK1	FRAS1	INPP4A	MITF	NUF2	PTP4A1	SHQ1	TOPBP1
ASXL1	CDX2	ERBB2	FUBP1	INPP4B	MKNK1	NUTM1	PTPN11	SIPA1	TP53
ATAD2	CEBPA	ERBB3	FYN	INSR	MLH1	NYAP2	PTPRD	SLC7A8	TP53BP1
ATF1	CETN2	ERBB4	G6PC	IRF2	MLH3	PAK1	PTPRO	SLC28A3	TP63
ATM	CFTR	ERCC1	GAB2	IRF4	MMS19	PAK5	PTPRS	SLC34A2	TPM3
ATR	CHD1	ERCC2	GABRA6	IRS2	MPL	PALB2	PTPRT	SLC45A3	TRAF2
ATRX	CHEK1	ERCC3	GALNT12	JAK1	MRE11	PARP1	QKI	SLCO1B1	TRAF7
AURKA	CHEK2	ERCC4	GATA1	JAK2	MS4A1	PARP2	RAB35	SLX1A	TRRAP
AURKB	CIC	ERCC5	GATA2	JAK3	MSH2	PARP3	RAC1	SLX4	TSC1
AXIN1	CLK2	ERCC6	GATA3	JMJD1C	MSH3	PARP4	RAC2	SMAD2	TSC2
AXIN2	COL11A1	ERF	GATA4	JUN	MSH4	PAX5	RAD21	SMAD3	TSHR

AXL	COL22A1	ERG	GATA6	KDM5C	MSH5	PAX8	RAD50	SMAD4	TUBB3
B2M	COP1	ERRFI1	GEN1	KDM6A	MSH6	PBRM1	RAD51	SMARCA1	TYMS
BABAM2	CREB1	ESR1	GGH	KDR	MSI1	PBX1	RAD51B	SMARCA4	U2AF1
BACH1	CREBBP	ETV1	GID4	KEAP1	MSI2	PCDH9	RAD51C	SMARCB1	UGT1A1
BAP1	CRKL	ETV4	GLI1	KIAA1549	MST1	PDCD1	RAD51D	SMARCD1	UMPS
BARD1	CSDE1	ETV5	GNA11	KIF1B	MST1R	PDCD1LG2	RAD52	SMO	UNC5D
BCL2	CSF1R	ETV6	GNAQ	KIF5B	MTAP	PDGFRA	RAD54B	SMYD3	UPF1
BCL2A1	CSMD3	EWSR1	GNAS	KIT	MTDH	PDGFRB	RAD54L	SNCAIP	USP6
BCL2L1	CTCF	EXO1	GPS2	KLF6	MTHFR	PDK1	RAF1	SOCS1	VEGFA
BCL6	CTLA4	EXOC2	GRB7	KLHL6	MTOR	PGR	RARA	SOD2	VHL
BCOR	CTNNA1	EXT1	GREM1	KLLN	MTRR	PHF6	RASA1	SOS1	VTCN1
BCR	CTNNB1	EXT2	GRIN2A	KMT2A	MUC6	PHOX2B	RB1	SOX2	WEE1
BIRC2	CTNND2	EZH1	GRM3	KMT2B	MUC16	PIK3CA	RBBP8	SOX4	WRN
BIRC3	CUL3	EZH2	GSK3B	KMT2C	MUS81	PIK3CB	RBM10	SOX9	WT1
BLM	CUL4A	EZR	GSTP1	KMT2D	MUTYH	PIK3CG	RECQL	SOX10	WWTR1
