# Supplementary material for: Neurosarcomatous amelanotic transformation of malignant melanoma presenting as malignant periopheral nerve sheath tumor: Rare case report
Source: Medicine (Baltimore). 2023 Jun 23;102(25):e34034. doi: 10.1097/MD.0000000000034034 (PMC10289641; doi:10.1097/MD.0000000000034034)
Supplement: Supplementary file 2 [file medi-102-e34034-s002.pdf]

Table 2 List of Microsatelliate (MSI) site

BAT25	BAT26	BAT34c4	BAT40	D17S261	D17S799	D18S35	D18S55	D18S58	D1S2883
D2S123	D5S346	MONO27	NR21	NR24					
